# On the apparent decrease in Olympic sprinter reaction times

**DOI:** 10.1371/journal.pone.0198633

**Published:** 2018-06-27

**Authors:** Payam Mirshams Shahshahani, David B. Lipps, Andrzej T. Galecki, James A. Ashton-Miller

**Affiliations:** 1 Department of Mechanical Engineering, University of Michigan, Ann Arbor, Michigan, United States of America; 2 School of Kinesiology, University of Michigan, Ann Arbor, Michigan, United States of America; 3 Institute of Gerontology, University of Michigan, Ann Arbor, Michigan, United States of America; 4 Department of Biostatistics, University of Michigan, Ann Arbor, Michigan, United States of America; University of Rome, ITALY

## Abstract

Reaction times of Olympic sprinters provide insights into the most rapid of human response times. To determine whether minimum reaction times have changed as athlete training has become ever more specialized, we analyzed the results from the Olympic Games between 2004 and 2016. The results for the 100 m and 110 m hurdle events show that minimum reaction times have systematically decreased between 2004 and 2016 for both sexes, with women showing a marked decrease since 2008 that eliminated the sex difference in 2012. Because overall race times have not systematically decreased between 2004 and 2016, the most likely explanation for the apparent decrease in reaction times is a reduction in the proprietary force thresholds used to calculate the reaction times based on force sensors in starting blocks—and not the result of more specialized or effective training.

## Introduction

The ability to rapidly respond to an external auditory stimulus is important when encountering emergent situations in daily activities such as driving or when operating machinery. Reaction times of sprinters at the Olympic Games offer insights into the fastest human reaction times because there is little question as to their states of arousal, motivation, learning or training [1]. (To avoid confusion, since this paper employs International Association of Athletics Federation (IAAF) data on ‘reaction time’, that term is used throughout this paper rather than the more usual scientific term ‘response time’.) It is unknown if the reaction times of sprinters at the Olympic Games remain stable from year to year, or whether changes to the focused training that athletes undergo in preparation for each Olympics can improve their reaction times. Understanding the effect of this focused training on the fastest human reaction times could provide insights into the trainability of athletes and other individuals for time-critical situations.

Investigating potential sex differences in the fastest human reaction times in elite Olympic sprinters has important implications on the design of human-machine interfaces for handling time-critical behaviors. Such interfaces, as in the braking system of an automobile, often do not consider potential sex differences in reaction times despite women having faster auditory latencies [2,3], shorter neural pathways [4] but less muscle strength [5]. A significant sex difference was found in the reported reaction times of sprinters at the 2008 Beijing Olympics Games, but this may have been an artifact of the algorithm used to calculate the reaction times [6]. One goal of this paper was to determine whether sex differences in reported reaction times have occurred at other Olympic Games. A second goal was to determine whether reaction times have decreased over Olympic years.

To measure reaction times on the Olympic Track Swiss Timing, a subsidiary of Omega SA, uses their ASC3 (Automatic Start Control) false start detection system which includes instrumented starting blocks to measure the time course of the force applied by the sprinter to the blocks with a precision of 1 ms following the starting gun. If this force, which their datasheet shows can reach ~2 kN, exceeds a given (unpublished) force threshold before 100 ms has elapsed from the onset of the start gun signal, a false start is registered (Rule 162, IAAF Competition Rules). A reaction time is reported in the published Olympic results if the force crosses the designated threshold after 100 ms, but no reaction time is reported in the event of a false start (< 100 ms) or other reasons for disqualification.

Any longitudinal comparisons of reaction time of sprinters competing at the Olympic Games between 2004 and 2016 is confounded by an IAAF rule change in 2010 (Rule 162.7, IAAF Competition Rules) that disqualified any athlete who false started, rather than permitting a second chance as the prior rule allowed. This change apparently led runners to adopt slightly more conservative strategies in their reaction times in order to avoid disqualification [7]. However, since there was no rule change between 2004 and 2008 or between 2012 and 2016, the results from those years should permit a direct comparison of athlete reaction times independent of the rule change.

## Methods

Official reaction times from every heat in the 100, 110, 200, 400 and 440 m track events in 2004, 2008, 2012 and 2016 were downloaded from the official IAAF web site. All names were stripped from the record to blind the analyses and University of Michigan Institutional Review Board Approval was received (Exempt—Not Regulated Research, HUM00135664, dated 9/8/2017). Runners who were disqualified in a heat were excluded, as were those who did not start. The reaction time (RT) data were positively skewed, so a power transformation (RT^-1.5^) was used to obtain a normal distribution [6].

Since we are interested in the minimum human auditory reaction times, we focused the analysis on the races with the shortest reaction times: in an initial analysis these proved to be the 100 m and 100 and 110 m hurdles races (S2 Fig and S1 Table). Since one reaction time was an outlier, exceeding 300 ms, we excluded it for being non-competitive; whether or not it was included in the analyses would prove not to affect the results.

All data analyses were performed in R (a language and environment for statistical computing) version 3.4.2. Linear mixed-effect models (LMM) with a random intercept for each athlete were fit to the data using the ‘nlme’ version 3.1–132 package. We allowed for different residual variations for each Olympic year and sex. Likelihood Ratio and t-tests were used to examine the roles of sex and year on an individual’s reaction time for the years 2004–2016. We only considered the minimum reaction time for each athlete for a given Olympic year; Those results were then back transformed to find the mean value as well as the -3SD minimum reaction time values (S1 Text) by sex and year [6].

**Fig 1:**
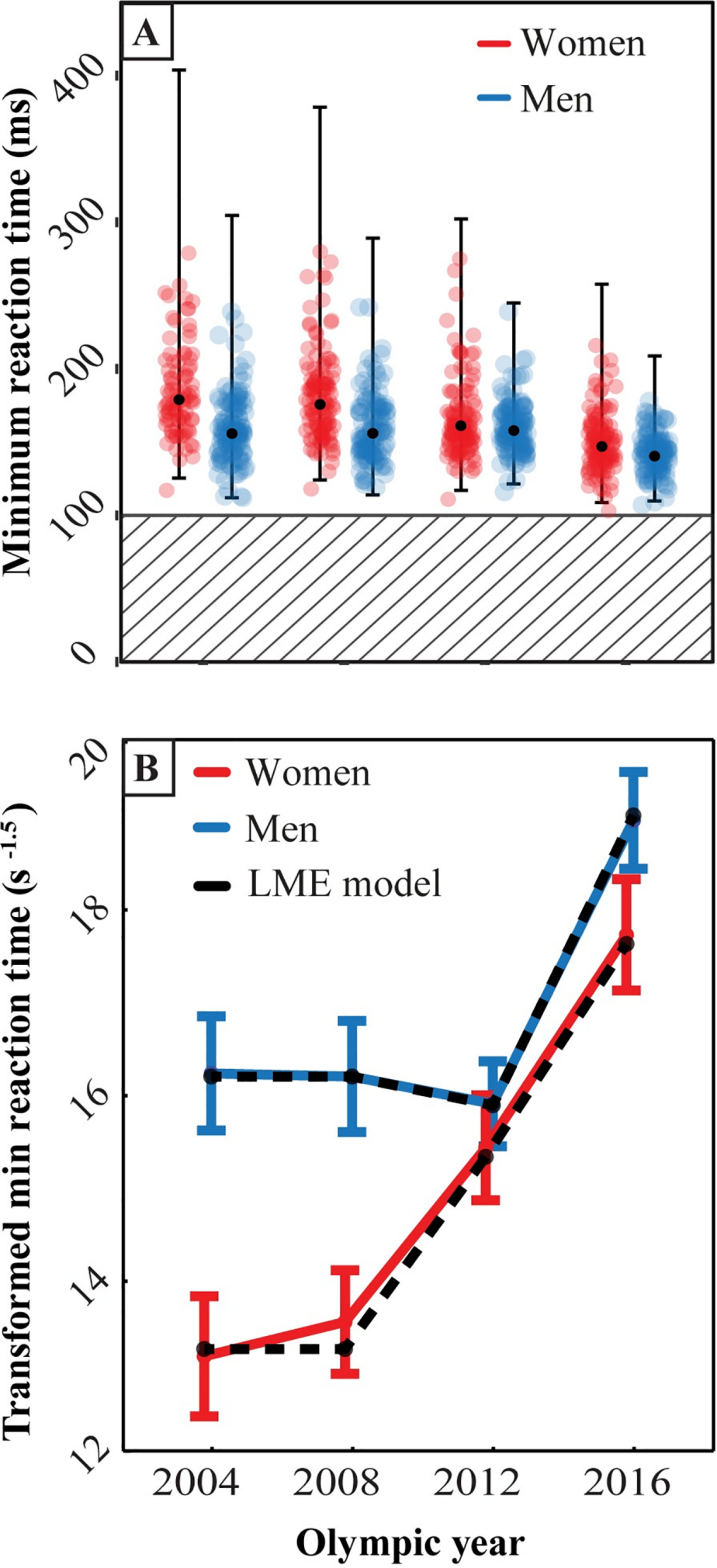
Sprinter minimum reaction times by sex and year in the 2004–2016 Olympics. (A) Scatter plot of reaction times by sex and year. The solid black circles and bars represent the mean and ±3SD reaction times after back-transformation. The hatched area designates reaction times deemed by IAAF rule to be a false start. The number of false starts reported for the 100 m sprints, 100 m hurdles, and 110 m hurdles were: 1 false start in 2004 and 2008 each, 3 in 2012, and 2 in 2016. (B) Mean (±2SE) transformed minimum reaction times (s^-1.5^) by sex and year. The 100 ms false start threshold when transformed becomes 31.6 s^-1.5^. The most parsimonious Linear Mixed-effect Model found to fit the data used a random intercept for each athlete. The fixed effects consisted of Olympic year as a factor with 3 levels (2004 and 2008 together, 2012, and 2016), Sex as a factor with 2 levels, with the interaction of Olympic year by sex. As a check, the predicted mean values from the LMM were found (the black dots and dashed lines) and agreed well with the original data (red and blue lines). Minimum reaction times decreased significantly by year (See Results section). Note that the 2010 IAAF rule change decreed that any runner who false started would be disqualified from the race, and that applied to the 2012 and 2016 Olympics.

Official reaction time results for the athletes who competed in the 2004, 2008, 2012 and 2016 Olympic sprinting competitions were included in our analyses (Fig 1A). To account for repeated measures a mixed-effect model with Olympic year and sex (4- and 2-level factors, respectively) was fit to the set of transformed minimum reaction times. A Likelihood Ratio (LR) test revealed a significant interaction between Olympic year and sex [LR = 22.82, p < 0.0001]. After considering the plots of the transformed minimum reaction times (Fig 1B), the mixed effect model was simplified based on the results of likelihood ratio tests for nested models. In particular the model was simplified by combining the 2004 and 2008 years into one category both in main effect of year as well as in year by sex interaction terms [LR = 1.20, p = 0.54].

## Results

The coefficients for the resulting fixed effects can be seen in Table 1 and, when considered with the results in Fig 1B, they suggest that reaction times decreased significantly in the more recent Olympics. When only the 2012 and 2016 data were considered as the fixed effects in the model, there remained significant year and sex differences, but the interaction was no longer significant [LR = 2.54, p = 0.111]. This suggests that the large sex differences in 2004 and 2008, which disappeared in 2012, drove the significant interaction term.

**Table 1:** Linear mixed-effect model for transformed minimum reaction times (s^-1.5^).

Fixed effect	Parameter estimate (s^-1.5^)	SE	p
*Olympic year (2004*, *2008)*	13.28	0.22	<0.001
*Olympic year (2012)*	15.37	0.28	<0.001
*Olympic year (2016)*	17.68	0.3	<0.001
*Olympic year (2004*, *2008)*:*Men*	3.05	0.31	<0.001
*Olympic year (2012)*:*Men*	0.51	0.35	0.16
*Olympic year (2016)*:*Men*	1.3	0.39	0.001

The analysis shows that women’s reaction times decreased significantly each year from 2008 to 2012, and 2012 to 2016. However, men’s reaction times only decreased significantly from 2012 to 2016 (Fig 1B).

## Discussion

Our starting point for this study was the finding by Lipps *et al*. [6] of a significant sex difference in the reaction times of sprinters at the 2008 Beijing Olympics. Natural questions were whether this difference would persist at the 2012 London Olympics and whether ever more focused training leads to slight decreases in reaction times for both men and women. The present results showing the abrupt nature of the decrease for only women at the London Olympics suggests something other than training may have been responsible. One explanation for the apparent decrease in minimum reaction times from 2012 to 2016 is that both sexes became more comfortable competing under the threat of disqualification imposed by the 2010 IAAF rule change, and thereby became less conservative. If so, the results do not support this explanation because there is no systematic decrease in the race finish times between 2012 and 2016 (S1 Fig). A simpler explanation is that the decreasing reaction times between 2012 and 2016 might be due to a decrease in the force thresholds employed by Swiss Timing to calculate the reaction times of the men and women [8]. It was suggested by Lipps *et al*. that a 22% decrease in the force threshold could have eliminated the sex difference in the reported 2008 reaction time data [6]. The present data for the 2012 Olympics suggest that the force threshold was indeed reduced for the women in 2012, but not the men; there was no significant sex difference in their reported reaction times, and the reaction times of the men did not change between 2008 and 2012 (Table 1, Fig 1B). There is no physiologic reason why women should be slower than men in their central auditory processing time [2]; put simply, their shorter limbs mean the signal reaches the leg muscles more quickly than in men, but their smaller leg muscles mean than it takes longer to develop a significant plantarflexion force against the starting block [6]. This means that it may be fruitful for companies to re-examine how they detect a false start with an automatic starting system. Our results suggest that the back-transformed Mean - 3SD value should be set to 100 ms (S1 Text, Fig 1A).

The method for calculating reaction time can vary with the company awarded the timing contract [9], so such a reduction is within the purview of Swiss Timing, who regard the force threshold used as proprietary information [6]. The choice of this force threshold is an important compromise for the quality of the athletic competitions. If the force threshold is set too low, the slightest twitch could result in a false start being recorded by the IAAF-certified Start Information System (SIS). This would not be practical because too many sprinters would be disqualified and that would spoil the competition. If the force threshold is set too high, sprinters would exhibit unreasonably long reaction times. Of course, in the most recent IAAF rules, it is the starter that makes the final disqualification decision based on data from the SIS system as well as whether the athlete initiated his/her starting action before the starter pulled the trigger. Any motion that is not part of the continuous starting movement would simply result in a caution the first time (Rule 162.7-Note (i), IAAF Competition Rules). The IAAF has been examining SIS methods for detecting false starts. (see for example [10])

A limitation of this study is the absence of data for reaction times less than 100 ms because the reaction times are not reported for false-starts. This, and not having access to the starting block force-time curves, prevents an accurate calculation of the minimum human reaction time for lower force thresholds than were used; in those cases reaction times could become less than 100 ms.

We conclude that the apparent decrease in sprinter reaction times between 2004 and 2016 is caused by decreases in the force thresholds used to calculate the reaction time. The decrease in both men’s and women’s reaction times after 2012 appears to reflect fine tuning of those force thresholds by Swiss Timing rather than a decrease in the acoustic neuromuscular reaction time of the athletes due to specialized training.

In terms of applicability of the results to other situations, a rapid acoustic reaction time can be important, for example, when a driver needs to brake an automobile in an emergency after hearing a warning horn. Our results suggest that the designer of mechanical or electronic equipment to which humans are to be mechanically coupled should employ the lowest practical force threshold that does not disadvantage women.

## Conclusions

We conclude that the apparent decrease in reaction times is due to a reduction in the proprietary force thresholds used to calculate the reaction times, based on measurements from force sensors in the starting blocks, not the result of more specialized or effective training.

## Supporting information

S1 Text Why the Mean– 3SD value is a good estimate of minimum auditory reaction time for Olympic false start detection in sprinting. (DOCX)

S1 Fig Distribution of overall ‘mark’ times by sex, year, and race type. IAAF terminology designates the overall race time as the ‘mark’ time. The boxplot lines represent the median, and the first and third quartiles. The vertical lines extend up to 1.5 times the interquartile distance from the top and bottom boxplot lines. The graph shows no systematic change in overall race times with Olympic year for men or women.
(TIF)

S2 Fig Transformed minimum reaction times for all the sprints in 2016. Distribution of transformed minimum (min) reaction time in 2016 for 196, 46, 63, 148, 138, and 94 athletes who competed in 100 m, 100 m hurdles, 110 m hurdles, 200 m, 400 m, and 400 m hurdles respectively. The error bars show ±2SE. The shorter sprints (100 m, 100 m hurdles, and 110 m hurdles) have significantly faster reaction times than the longer sprints (p = 0.0001). To view the linear mixed effect model for this analysis, please refer to S1 Table.
(TIF)

S1 Table Linear mixed-effect model results for transformed minimum reaction times for all sprints in 2016. Transformed minimum reaction time results for the 100 m sprints was chosen as the reference group. We can see that 100 m hurdles, and 110 m hurdles were not significantly different than the reference. However, 200 m, 400 m, and 400 m hurdles were significantly different than the 100 m sprints.
(PDF)
