# Supplementary material for: On the apparent decrease in Olympic sprinter reaction times
Source: PLoS One. 2018 Jun 27;13(6):e0198633. doi: 10.1371/journal.pone.0198633 (PMC6021049; doi:10.1371/journal.pone.0198633)
Supplement: S1 Text — (DOCX) [file pone.0198633.s001.docx]

S1 Text: Why the Mean – 3SD value is a good estimate of minimum auditory reaction time for Olympic false start detection in sprinting.

There are 14 IAAF Diamond League events for the best sprinters in the world to compete in each year. If a sprinter qualifies for all three heats in both the 100 and 200 m events, that would mean 14 x 3 x 2 starts/year (not counting the relays). Whether an Olympic year or an off year, the 3 x 2 starts for the national team selection and the 3 x 2 starts in the Olympics or World Championships starts should then be added to these resulting in a total of 96 starts/year. If the force threshold was to be set for each sex such that the mean – 3SD point after back-transformation was 100 ms, then one might expect an athlete to false start in 1/0.00135 races or 740 races or ~8 years of racing. To our knowledge, Usain Bolt’s false start in the final of the Aug 29, 2011 World Championships was the only false start we could find reported for his 10 year career at major meets. This would be in agreement with using a Mean – 3SD criterion (Table 1) for determining the minimum human reaction time.

**Table A. Comparison of Mean – 3SD and Mean – 4SD values (in ms) by sex and year.**

|  | **Women** | | | | **Men** | | | |
| --- | --- | --- | --- | --- | --- | --- | --- | --- |
|  | **2004** | **2008** | **2012** | **2016** | **2004** | **2008** | **2012** | **2016** |
| **Mean - 3SD** | 125 | 124 | 117 | 108 | 112 | 113 | 124 | 110 |
| **Mean - 4SD** | 115 | 114 | 108 | 100 | 103 | 105 | 116 | 103 |

Note that one outlier was removed from the data for women in 2012 because it was deemed a non-competitive reaction time (> 300 ms).
