# Supplementary figures and images for: On the apparent decrease in Olympic sprinter reaction times
Source: PLoS One. 2018 Jun 27;13(6):e0198633. doi: 10.1371/journal.pone.0198633 (PMC6021049; doi:10.1371/journal.pone.0198633)

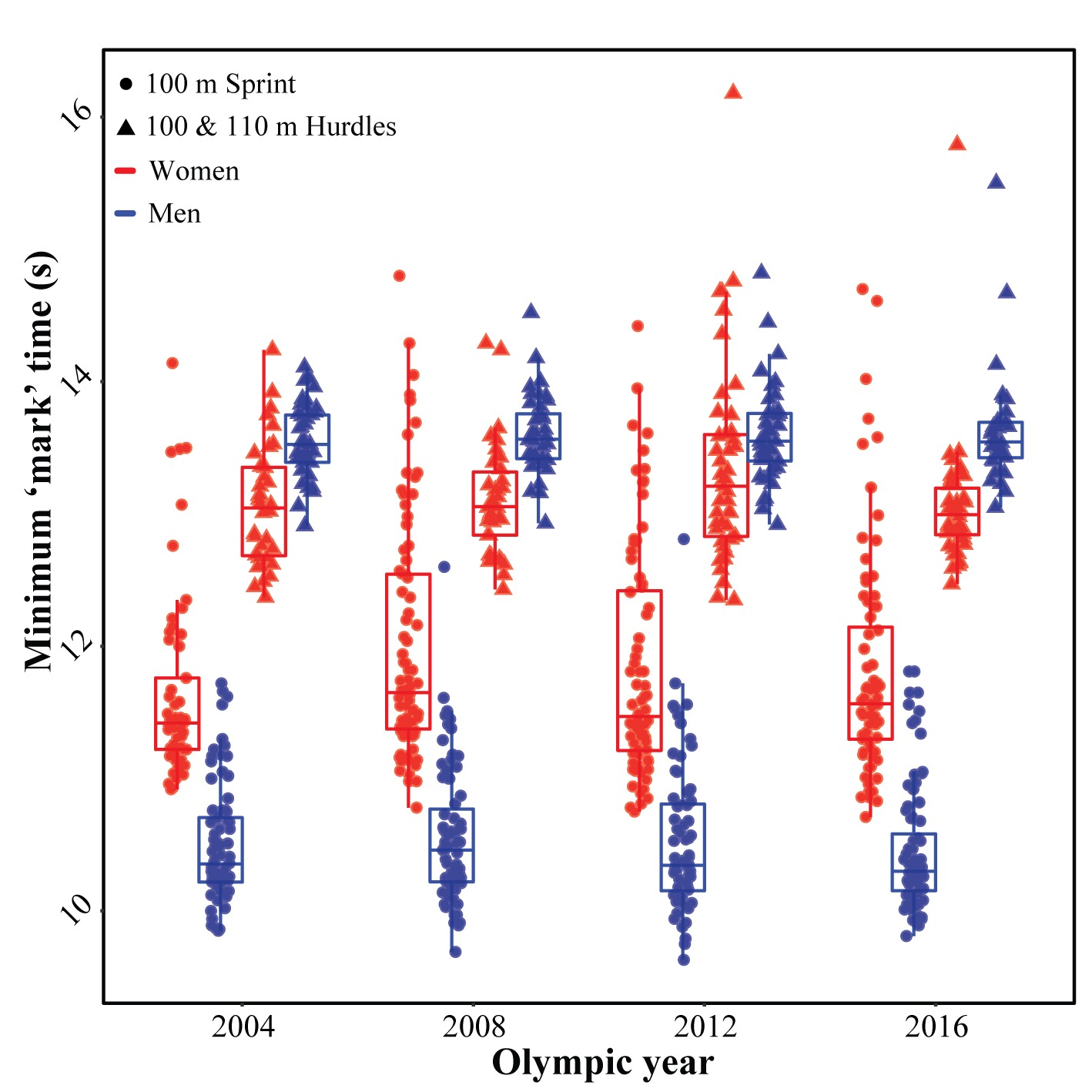

Supplement: S1 Fig — IAAF terminology designates the overall race time as the ‘mark’ time. The boxplot lines represent the median, and the first and third quartiles. The vertical lines extend up to 1.5 times the interquartile distance from the top and bottom boxplot lines. The graph shows no systematic change in overall race times with Olympic year for men or women. (TIF) [file pone.0198633.s002.tif]
