# Supplementary material for: On the apparent decrease in Olympic sprinter reaction times
Source: PLoS One. 2018 Jun 27;13(6):e0198633. doi: 10.1371/journal.pone.0198633 (PMC6021049; doi:10.1371/journal.pone.0198633)
Supplement: S1 Table — Transformed minimum reaction time results for the 100 m sprints was chosen as the reference group. We can see that 100 m hurdles, and 110 m hurdles were not significantly different than the reference. However, 200 m, 400 m, and 400 m hurdles were significantly different than the 100 m sprints. (PDF) [file pone.0198633.s004.pdf]

Table A. Linear mixed-effect model results for transformed minimum reaction times for all sprints in 2016.

Fixed effect	Parameter estimate ($s^{-1.5}$)	SE	p
(Intercept)	17.860	0.214	< 0.001
100 m H	0.290	0.552	0.599
110 m H	0.161	0.394	0.684
200 m	-1.304	0.324	< 0.001
400 m	-2.514	0.333	< 0.001
400 m H	-2.847	0.452	< 0.001
